# Supplementary material for: A Humanized Yeast Phenomic Model of Deoxycytidine Kinase to Predict Genetic Buffering of Nucleoside Analog Cytotoxicity
Source: Genes (Basel). 2019 Sep 30;10(10):770. doi: 10.3390/genes10100770 (PMC6826991; doi:10.3390/genes10100770)
Supplement: Supplementary file 1 [file genes-10-00770-s001.zip › Additional_Files/Additional_File7_GOTermHeatmaps.zip!/Additional_File7_GOTermHeatmaps/perinuclear_region_of_cytoplasm.pdf]

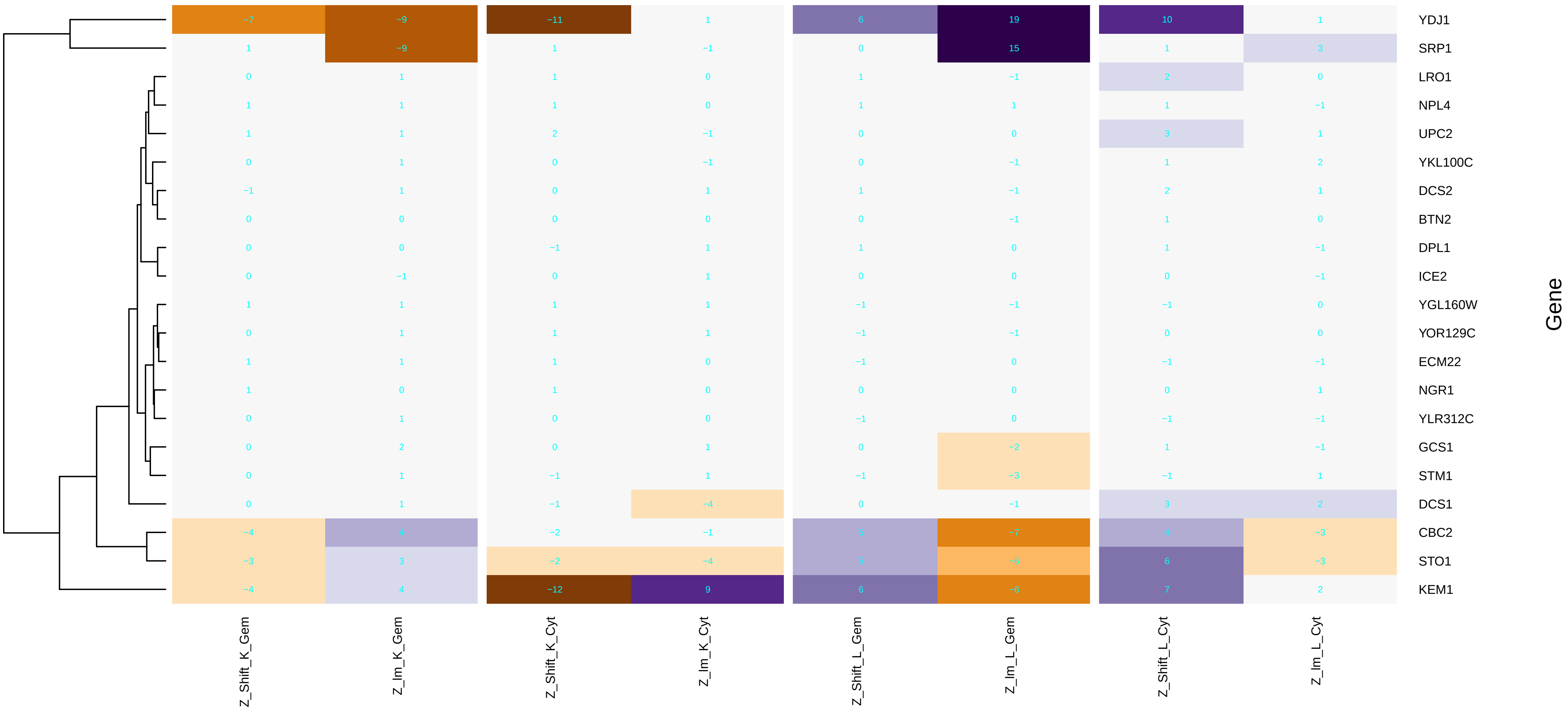
YDJ1 −7 −9 −11 1 6 19 10 1
SRP1 1 −9 1 −1 0 15 1 3
LRO1 0 1 1 0 1 −1 2 0
NPL4 1 1 1 0 1 1 1 −1
UPC2 1 1 2 −1 0 0 3 1
YKL100C 0 1 0 −1 0 −1 1 2
DCS2 −1 1 0 1 1 −1 2 1
BTN2 0 0 0 0 0 −1 1 0
DPL1 0 0 −1 1 1 0 1 −1
ICE2 0 −1 0 1 0 0 0 −1
YGL160W 1 1 1 1 −1 −1 −1 0
YOR129C 0 1 1 1 −1 −1 0 0
ECM22 1 1 1 0 −1 0 −1 −1
NGR1 1 0 1 0 0 0 0 1
YLR312C 0 1 0 0 −1 0 −1 −1
GCS1 0 2 0 1 0 −2 1 −1
STM1 0 1 −1 1 −1 −3 −1 1
DCS1 0 1 −1 −4 0 −1 3 2
CBC2 −4 4 −2 −1 5 −7 4 −3
STO1 −3 3 −2 −4 5 −6 6 −3
KEM1 −4 4 −12 9 6 −6 7 2
Z_Shift_K_Gem
Z_Im_K_Gem
Z_Shift_K_Cyt
Z_Im_K_Cyt
Z_Shift_L_Gem
Z_Im_L_Gem
Z_Shift_L_Cyt
Z_Im_L_Cyt
Gene
